# Prognosis of Bearing and Gear Wears Using Convolutional Neural Network with Hybrid Loss Function

**DOI:** 10.3390/s20123539

**Published:** 2020-06-22

**Authors:** Chang-Cheng Lo, Ching-Hung Lee, Wen-Cheng Huang

**Affiliations:** 1 Department of Mechanical Engineering, National Chung Hsing University, Taichung 402, Taiwan; hchs.jason910657@gmail.com; 2 Microprogram Information Co., Ltd., Taichung 407, Taiwan; wencheng@program.com.tw

**Keywords:** wear prognosis, deep learning, convolutional neural network, vibration signal

## Abstract

This study aimed to propose a prognostic method based on a one-dimensional convolutional neural network (1-D CNN) with clustering loss by classification training. The 1-D CNN was trained by collecting the vibration signals of normal and malfunction data in hybrid loss function (i.e., classification loss in output and clustering loss in feature space). Subsequently, the obtained feature was adopted to estimate the status for prognosis. The open bearing dataset and established gear platform were utilized to validate the functionality and feasibility of the proposed model. Moreover, the experimental platform was used to simulate the gear mechanism of the semiconductor robot to conduct a practical experiment to verify the accuracy of the model estimation. The experimental results demonstrate the performance and effectiveness of the proposed method.

## 1. Introduction

In industry, production lines are required to be automated and operate more stably for the quality of products. With early prognosis, the manufacturer can schedule downtime maintenance more efficiently. Many studies have been proposed on the diagnosis and prognosis of mechanical parts such as bearings, gears, and motors [1,2,3,4]. The study in [2] proposed a current signal analysis method by empirical mode decomposition and the Hilbert spectrum for the incipient broken rotor of induction motors. Through statistical analysis, the damage was detected by the kurtosis value in early phases. Xiaohang Jin et al. used the health index obtained from preprocessing the data to detect early faults of bearing that demarcate the remaining useful life (RUL) [3]. Additionally, a motor current signature analysis method for gear wear monitoring has been proposed based on the modulation signal bispectrum [4]. The monitoring process was implemented on the current signals from a run-to-failure test on helical gearbox accelerated fatigue.

In recent years, data-driven technologies are glowing with the popularity of automation and the convenience of data acquirement. Data-driven technologies make heavy use of artificial intelligence and machine learning to diagnose through larger amounts of data analysis and learning [1,5]. It does not need complex modeling and can intelligently improve the diagnosis accuracy by adaptively learning. Guo et al. proposed a recurrent neural network based health indicator for the RUL prediction of bearings [6], which is proposed to map the vibration signal features from 0 to 1 through a recurrent neural network (RNN) and the double exponential model was introduced to predict the bearing RUL. Furthermore, a variety of deep learning researches has been proposed recently [7,8,9,10] and show that through training on data, the features can be automatically extracted by the deep learning model instead of manual extraction. These studies require full-time monitoring to collect the corresponding data; however, obtaining wear data for the complete process is difficult. On the other hand, it is much easier to collect the data of normal and malfunctioning samples, but the original model cannot achieve estimation as there are only classification labels for data.

According to the research proposed by Erxue Min et al., they surveyed the research of clustering with deep learning, and showed that the models could extract clustering features by training with designed clustering loss [11]. Moreover, Elie Aljalbout et al. proposed a taxonomy of clustering methods of deep neural networks [12]. Deep learning models were trained on both non-clustering loss and clustering loss to suit their tasks. It has also been shown that vibration signals manifest the status of the machine in the time domain, frequency domain, and time–frequency domain [1,13,14,15,16,17]. In this paper, a deep learning model with clustering loss was proposed for vibration signals, and proper features for clustering were extracted through training. The extracted features were subsequently used to estimate the current wear status through raw vibration signals. The proposed approach was applied to the open bearing dataset, and an established gear platform was utilized to validate the functionality and feasibility of the proposed model. Finally, the experimental platform was used to simulate the gear mechanism of the semiconductor robot to conduct a practical experiment to verify the accuracy of model estimation.

The rest of this paper is organized as follows. The proposed method is introduced in Section 2. Section 3 introduces the first experiment is a preliminary validation by the open bearing dataset and the gear experimental platform is introduced to evaluate the proposed method in practical problem in Section 4. Finally, the conclusions are given in Section 5.

## 2. One-Dimensional Convolutional Neural Network (1-D CNN) with Clustering Loss for Prognosis

This section provides an introduction to the deep neural network data-driven technology and the approach of establishing a one-dimension convolutional neural network (1-D CNN) model. The characteristics of this approach are suitable for the time-series concept and are subjoined by clustering loss. Finally, the estimation of wear with a simple linear function mapping is introduced. By monitoring the estimation continuously, the prognosis can be achieved.

### 2.1. One-Dimensional Convolutional Neural Network

**Figure 1:**
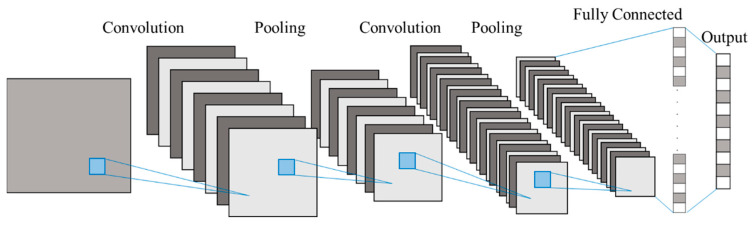
The architecture of the generally used convolutional neural network (CNN) [20].

Convolutional neural networks (CNNs) have been widely used in many image recognition systems, as shown in Figure 1 [18,19,20]. A CNN typically consists of convolutional layers, pooling layers, and a fully connected network. The convolutional layers contain many kernel filters that are used to catch the image features; the pooling layers have the ability of downsampling to obtain a lower resolution feature map. Subsequently, the final feature maps connect to fully connected layers. In the end, the model is trained to reduce the error between the network output and target output through the backpropagation algorithm. CNN can detect the information of hidden features from raw input inherently due to the reused kernel filters. Therefore, if the defect characteristic signals also occur in vibration signals repeatedly, then each defect characteristic signal is similar to each other.

**Figure 2:**
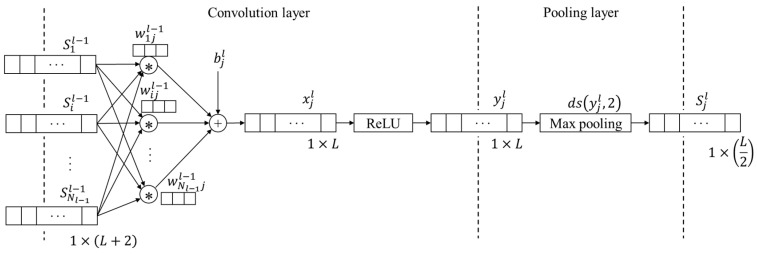
The one-dimensional convolutional neural network (1-D CNN) structure illustration: convolution and pooling operations [22].

A sequence of the one-dimensional CNN model, proposed by Turker Ince et al. [21], was applied in this study. They proposed a motor anomaly detection and condition monitoring system using an adaptive one-dimensional convolutional neural network (1-D CNN). The 1-D CNN structure is introduced in Figure 2 [22], and $x_{j}^{l}$ denotes the forward propagation from previous layer *l* − 1, in other words:

(1) $$x_{j}^{l}b_{j}^{l}+\sum_{i=1}^{N_{l-1}}conv\left(w_{ij}^{l-1},S_{i}^{l-1}\right)$$

where $b_{j}^{l}$ is the bias of *j*th neuron at current layer *l*; $w_{ij}^{l-1}$ is the kernel filter between *i*th neuron of layer *l* − 1 and *j*th neuron of layer *l*; $S_{i}^{l-1}$ is the output of *i*th convolution neuron at layer *l* − 1; and the output of the convolutional neuron $y_{i}^{l}$ is

(2) $$y_{j}^{l}=f\left(x_{j}^{l}\right)$$

At the pooling operation *ds*, the output $S_{j}^{l}$ is

(3) $$S_{j}^{l}=ds\left(y_{j}^{l},2\right)$$

As shown in Figure 2, the input length is *L* + 2 and the length of the sequence after the convolution operation is *L* because the kernel filter length is 3. The pooling operation, which down-samples the signal by a factor of 2, shows that the final length of output sequence should be *L*/2. After multiple layers of convolution layers and pooling layers are connected, the classifier or regressor is connected below through a flattened layer.

### 2.2. Clustering Loss

**Figure 3:**
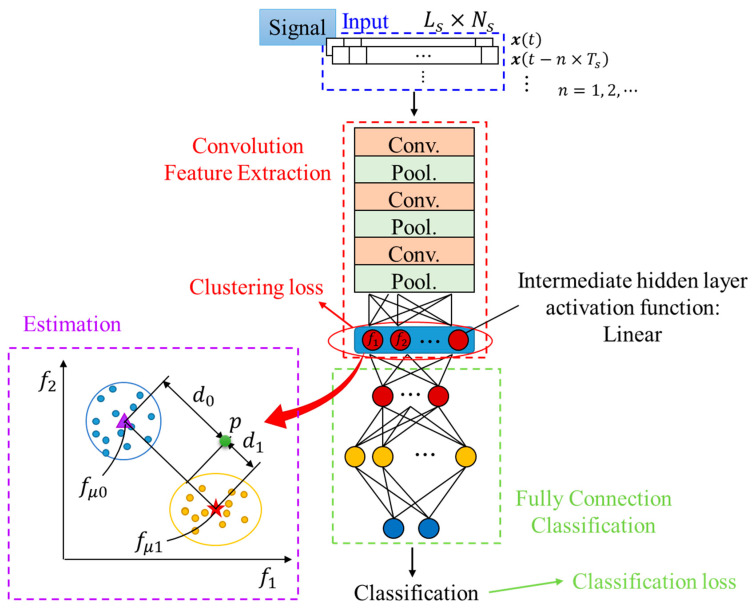
The whole 1-D CNN based on the clustering loss estimation model.

**Figure 4:**
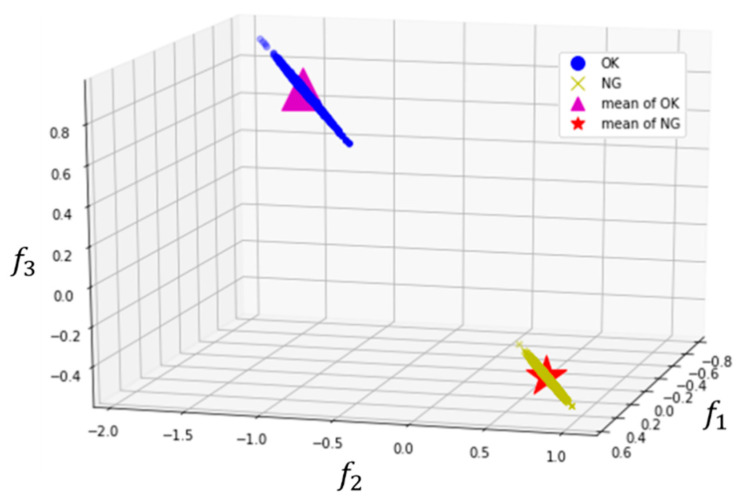
The clustering feature diagram.

In the first attempt to experiment with the data, the normal vibration signals were very similar to the failure samples and it was difficult to extract useful features by 1-D CNN alone. One can expect that as the wear progress develops, the extracted features would not only gradually change from normal (OK) to wear failure (NG), but are also clustered separately. For clustering the intermediate features of the hidden layer output extracted by 1-D CNN, clustering loss in feature space for training the 1-D CNN classification model is introduced, as shown in Figure 3, where blue, red, and green are the input, convolution, and classification, respectively. Furthermore, the proposed 1-D CNN model after training can extract clustering features, and the outputs from the hidden layer are added and trained in the whole model with both classification and clustering loss functions. This is based on the Euclidean distance of features in high dimensional feature space. Figure 4 shows the result of clustering features *f*_1_, *f*_2_, and *f*_3_ in the hidden layer, respectively; blue circles, yellow crosses, magenta triangle, and red star markers are the OK, NG, mean of OK, and mean of NG data distribution, respectively. As the feature outputs of OK data cluster well on one side, NG data cluster on the other side.

The purpose of clustering loss *L_cluster_* is to obtain group separation features (i.e., a large value of distance *D* and small *r*_0,_ *r*_1_,), where *D* denotes the distance of mean values $f_{\mu 0}$ and $f_{\mu 1}$ of OK and NG data:

(4) $$D=\sqrt{\sum_{n=1}^{N}{\left(f_{n}^{\mu ,0}-f_{n}^{\mu ,1}\right)}^{2}}$$

and *N*is the hidden layer feature output number; $f_{n}^{\mu ,0}$ is the *n*th average output of label 0 data; $f_{n}^{m}$ is the *n*th feature output of *m*th data; *M*_0_ is the number of label 0 data; and *r*_0_ and *r*_1_ are the cluster radiuses, in other words

(5) $$f_{n}^{\mu ,*}=\frac{1}{M^{*}}\sum_{m=1}^{M^{*}}f_{n}^{m},\;*=0,\;1$$

(6) $$r_{*}=\frac{1}{M^{*}}\sum_{m=1}^{M^{*}}\sqrt{\sum_{n=1}^{N}{\left(f_{n}^{m}-f_{n}^{\mu ,*}\right)}^{2}},\;*=0,\;1.$$

A two-dimension feature space illustration is also shown in Figure 3 of the estimation part. The clustering loss function is designed as

(7) $$L_{cluster}\left(D_{norm}\right)=\ln\left(\frac{1+e^{-\beta \left(x+1-\alpha \right)}}{1+e^{-\beta \alpha }}\right)$$

where

(8) $$D_{norm}\equiv \frac{D-\left(r_{0}+r_{1}\right)}{D}$$

**Figure 5:**
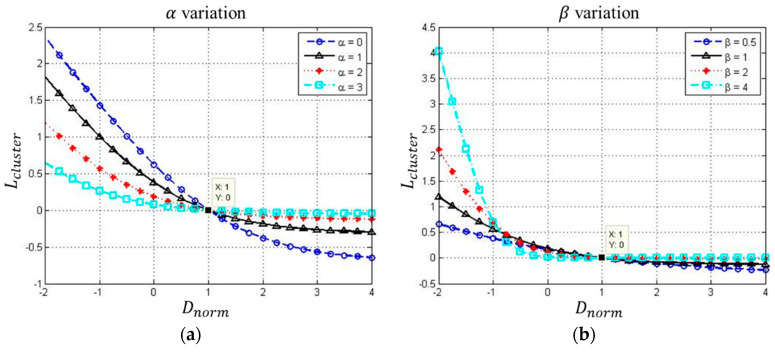
The visualized loss function curve, (**a**) *α* variation; (**b**) *β* variation.

*α* and *β* are the parameters of the loss function with *α* ≥ 0, *β* ≥ 1. With the aim to increase the distance between two clusters and reduce the dispersion of each cluster simultaneously, *D_norm_* was designed as a ratio variable instead of *D*, *r*_0_, and *r*_1_ directly. Then, an exponential loss function was designed for the gradual and smooth convergence of training. As *D_norm_* approached 1, loss approached zero. Figure 5 shows the curve of *L_cluster_*(*D_norm_*) with different values of *α* and *β.*

To minimize the loss function results in *D_norm_* approaches to 1, *D* would be much larger than (*r*_0_ + *r*_1_), which makes the feature outputs cluster. The reason for applying exponential function as a loss curve instead of a linear function is the smooth learning of the model, which learns from not only the clustering loss, but also the classification loss. It is necessary to adjust *α* and *β* moderately to prevent the model from overly learning against the clustering loss and results in highly sensitive estimating. The estimation approach is introduced in the next section.

### 2.3. Time Series Input

**Figure 6:**
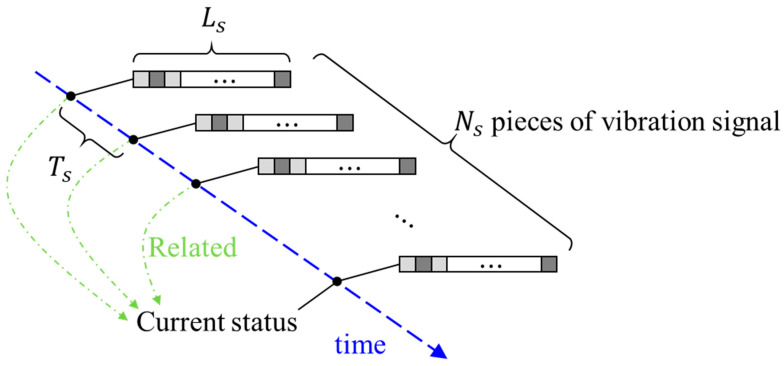
Illustration of time series inputs for 1-D CNN.

Wear is a gradual process caused by the damage from the removal of the material over time [23]. As a result, the current situation depends on the previous one. Therefore, a 1-D CNN model was introduced to take the vibration signals of both the current time and previous time as an input, instead of the signal of current time only. The corresponding 1-D CNN with clustering loss and time-series inputs is introduced in Figure 3, where the time-series input is shown in the blue block, and *L_s_* is the input sequence length; *N_s_* is the number of input sequences; *n =* 1, …, *N_s_*; and *T_s_* is the time-shift interval between each signal. A more detailed illustration is shown in Figure 6. Herein, the signal length *L_s_* must be greater than or equal to two times the length of the signal period. In addition, a larger value of *N_s_* results in a greater amount of calculation, and takes more time for data acquisition. Moreover, the time series interval would be meaningless since there is little variation of the signal if *T_s_* is too small.

### 2.4. The Proposed Approach for Prognosis Approach

Estimation by vibration signals can timely and continuously monitor the wear of mechanism components. Therefore, the proposed approach in this research is an estimation based on classification wear data: 1-D CNN with clustering loss was applied for further prognosis. As above, the 1-D CNN model was trained on the classification data with classification loss at the output layer and clustering loss at the intermediate hidden layer. After training by the proposed model shown in Figure 3, the proper feature was obtained in the flattened layer. Hence, a linear neuron layer weighting sum of these nodes of the flattened layer was designed as a fixed number of outputs. The wear amount was calculated by these features, an illustration of estimation is also shown in the estimation part of Figure 3, where *f_µ_*_0_ and *f_µ_*_1_ in feature space were obtained from the OK and NG data of the training data. When new data are obtained, the estimation system maps the input signals to feature outputs, shown as point *p*. Then, project *p* onto the line of *f_µ_*_0_ and *f_µ_*_1_, *d*_0_ and *d*_1_ can be obtained. Moreover, the variation in the location of *p* to a linear function is mapped. Final estimation wear amount *P* is calculated by

(9) $$P=W\left(\frac{d_{0}}{d_{0}+d_{1}}\right)$$

where *W* is the average wear amount of the NG sample.

## 3. Analysis and Validation: IEEE Prognostics and Health Management (PHM) Open Dataset

Bearings are essential mechanical parts and have operated for a long time as consumables, hence the wear of the bearings is quite considerable. In this section, an open bearing dataset was used for the experiment to preliminarily validate the proposed method. The lack of the entire wear process data was to simulate an actual manufacturing field and the data were labeled by the categorical RUL ratio. The effect of *L_s_*, *N_s_*, and *T_s_* were also analyzed on the 1-D CNN with clustering loss. The proposed method on the RUL estimation was verified by comparing the estimation error and functionality with other studies.

### 3.1. Data Acquirement and Processing

The open bearing dataset was obtained from the Institute of Electrical and Electronics Engineers (IEEE) Prognostics and Health Management (PHM) 2012 Prognostic Challenge [24]. It is a run-to-failure experiment and is an online health monitor through the accelerated degradation of bearings under adjustable operating conditions. The data gathered under three different loads (rotating speed and load force) contains rotating vibration, speed, load force, and the temperature of bearings. The sampling frequency was 25.6 kHz and the recording time was 0.1 s while the time interval of each piece of data was 10 s. Furthermore, six run-to-failure datasets were provided to build the prognostic models, 11 remaining bearings were used to evaluate the estimation accuracy of the bearings’ remaining useful life (RUL). In this experiment, the wear amount was replaced with the RUL of the bearings to simulate what manufacturers are lacking in the complete wear process data.

The RUL ratio of bearings was used as the target for the model estimation at first. The elapsed time of each piece of data was divided from the beginning by the total wear time of the bearing. Next, since the model requires time-series input, an arrangement of data is necessary, according to the *L_s_*, *N_s_*, and *T_s_* designed, *N_s_* pieces, *L_s_* length of the one axis vibration signal data, and the RUL ratio of the last signal is set as an input and output pair (training pattern). Note that it was assumed that the manufacturer acquires only a few vibration signals of and wear failure samples in this study. The arranged data whose RUL ratio greater than 0.75 and less than 0.25 would be treated as OK and NG data. To evaluate the estimation performance, the whole dataset of 17 bearings was kept with the original RUL ratio target as an estimation set.

Generally, the total dataset was divided into three parts: training, validation, and test sets. Therefore, for building the 1-D CNN classification model, the 80% data of six bearings were used for the training set, and 20% of the remaining data were used for the validation set; the data of 11 bearings were used for the test set, similar to the IEEE PHM challenge. Finally, the input data were normalized for preventing abnormal calculation values, and the vibration signal was re-scaled to within the range [−1, 1].

### 3.2. 1-D CNN with Clustering Loss Model Analysis

In this section, the training results and the effect of selected parameters *L_s_*, *N_s_*, and *T_s_* on the 1-D CNN with the clustering loss model are introduced. For a rotation speed of about 1500~1800 rpm, the characteristic defect frequencies of the bearing were higher than 25 Hz. The input signal length *L_s_* was selected as 2048 and the longest signal period of one cycle was determined as 1024 samples. There were 2560 sampling points in a single separated data as the maximum data length, so it is feasible for the *L_s_* to be consequently designed to 2048.

The proposed 1-D CNN shown in Figure 3 was adopted to treat the problem. Herein, the convolution of the first few layers does not stack with the pooling layer. After multiple layers of convolution, it overlaps with the pooling layer for reducing excessive calculations and outputs the hidden layer features *f*_1_, *f*_2_. The clustering loss was added to the intermediate hidden layer output calculation to make the features extracted from the OK and NG data have a clustering effect. Subsequently, the classifier part is to distinguish the eight hidden layer features of OK and NG data into two classes. Its structure is a fully connected simple neural network structure of [2,8,32] (one hidden layer). The final one is the estimation part. Since the data were pre-processed by dividing them by the 0.75 and 0.25 RUL ratio (i.e., the *f_µ_*_0_ and *f_µ_*_1_ was 0.875 and 0.125), the estimation value *P* is

(10) $$P=\left(0.875-0.125\right)\left(\frac{d_{0}}{d_{0}+d_{1}}\right)+0.125$$

**Figure 7:**
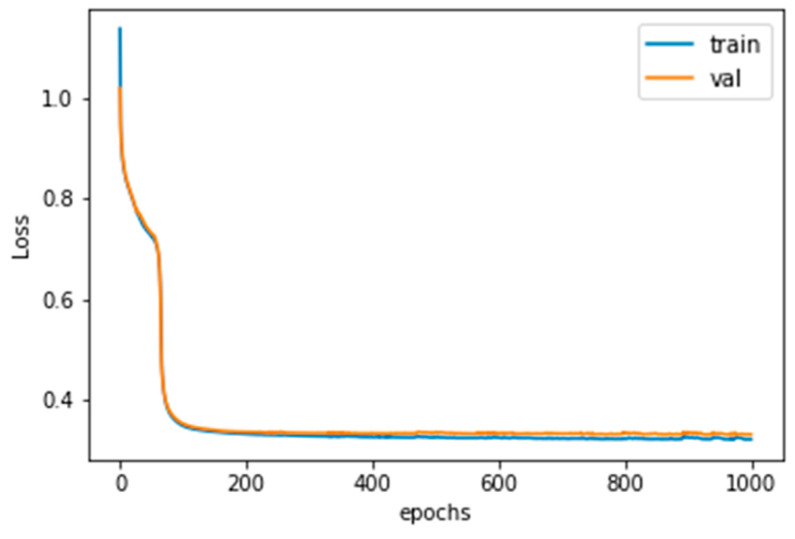
The training loss history.

The learning parameters designed are shown in Table 1; and the training result of the root mean square error (RMSE) is shown in Figure 7, blue: training loss; orange: validation loss. This shows that the training was successful and the overfitting phenomenon was not serious.

***Discussion 1: Learning Algorithm Selection***

The effects and results were compared to other popular algorithms in Table 2, which are the results of the same model trained on different algorithms. It can be seen that the use of Adam could obtain lower loss and higher accuracy under the same initial learning rate and epoch number. For the reasons of efficiency and convenience, Adam was selected as the training algorithm.

***Discussion 2: Time-Series Input Scheme***

**Figure 8:**
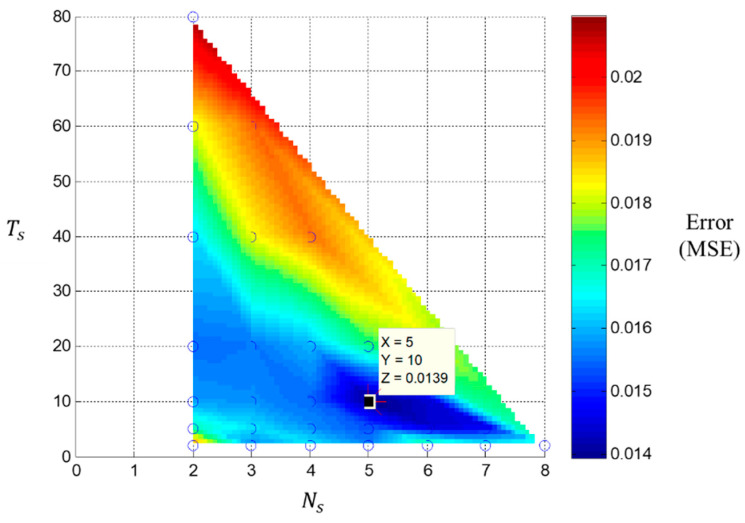
The training bearing remaining useful life (RUL) ratio estimation error comparison graph.

**Figure 9:**
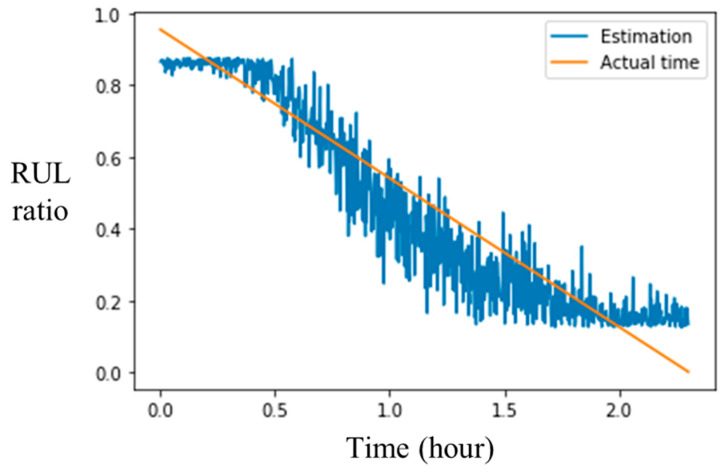
The continuous monitoring of the entire process data of the training set.

Herein, a comparison result of parameter analysis for the time-series input scheme is introduced in Table 3, where each trained model is depicted by (*N_s_*, *T_s_*). It can be observed that the classification ability is feasible since the accuracy of the training and validation set classification was almost 100% and the accuracy of the test set was greater than 80%. Then, the data of the training set bearings with the original RUL ratio target were used for comparing each model. The mean square error (MSE) of each model estimation is shown in Figure 8, where the color bar shows the corresponding MSE. The model (5, 10) had the minimum MSE 0.0139. Hence, the parameters (*N_s_*, *T_s_*) of 1-DNN with clustering loss designed were suggested as (5, 10). Moreover, the entire process data of the training sets were used for observing the continuous monitoring ability, which is shown in Figure 9, blue: estimation RUL ratio; orange: actual time of vibration signal data. The estimation RUL ratio gradually decreased over time.

***Discussion 3: CNN with Clustering Loss***

**Figure 10:**
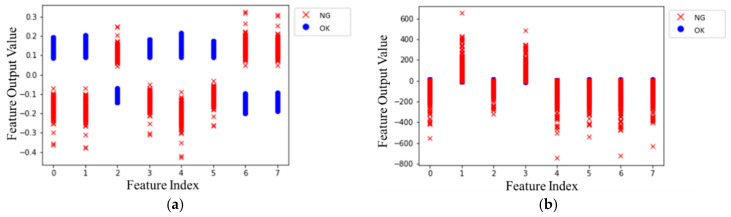
The features output clustering chart, (**a**) CNN with clustering loss; (**b**) CNN without clustering loss.

In addition, the corresponding feature after training is introduced in Figure 10, where ● and × denote OK and NG, respectively. There was a model trained without clustering loss for comparing the effect of clustering loss, confirming that the clustering loss is feasible for intermediate feature outputs clustering into each other. From Figure 10a, the CNN with clustering loss separated the features of both clusters; in contrast, the feature outputs overlapped and mingled messily with each other, as shown in Figure 10b. Simultaneously, Table 4 shows the cluster distance *D* and cluster radiuses *r*_0_, *r*_1_ of each cluster. Although distance *D* without clustering was larger than the model with clustering, the radiuses and *L_cluster_*(*D_norm_*) with clustering loss were smaller. Consequently, the clustering loss was effective for the feature outputs to cluster into each category.

## 4. Experimental Results: Gear Wear

To verify the proposed method, an experimental platform was designed to simulate one of the axes on a semiconductor robot arm.

### 4.1. Experimental Platform Setup

In this experiment, the gear wear problem of the robot was for transporting wafers in the semiconductor industry. To improve productivity and quality, robots operate stably for a long time in a vacuum environment. As a result, manufacturers have increased the maintenance standards and shutdown the robots frequently for repair, which increases production costs. However, the uncertainty between the samples of the robots was high, and there were also differences between the individual samples and the operating conditions, which makes the life of the robots different. Therefore, it is necessary to monitor the robot for a long time and determine whether it is malfunctioning. Furthermore, the robot arm is composed of a gear mechanism, and the vibration signal provides important information for the state of the mechanical part.

**Figure 11:**
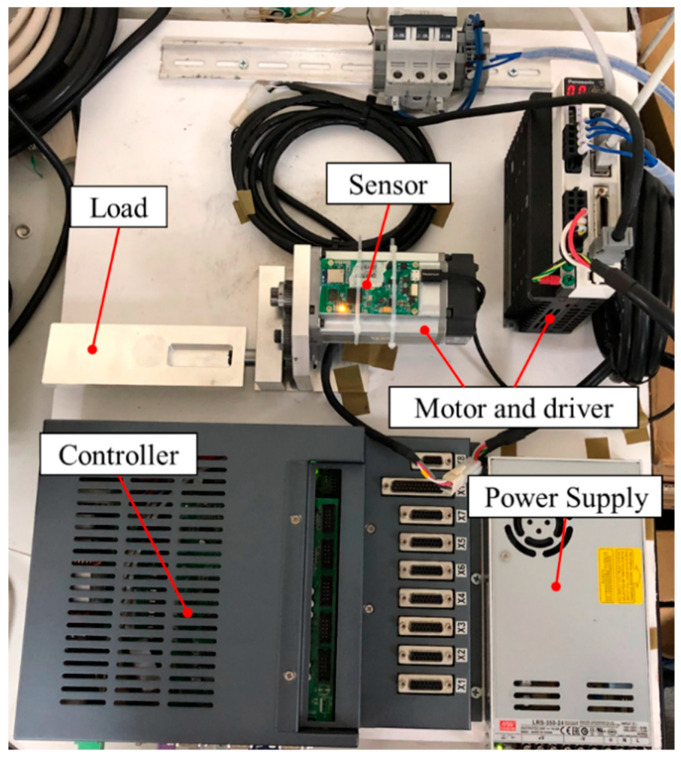
The platform of gear mechanism in National Chung Hsing University (NCHU).

**Figure 12:**
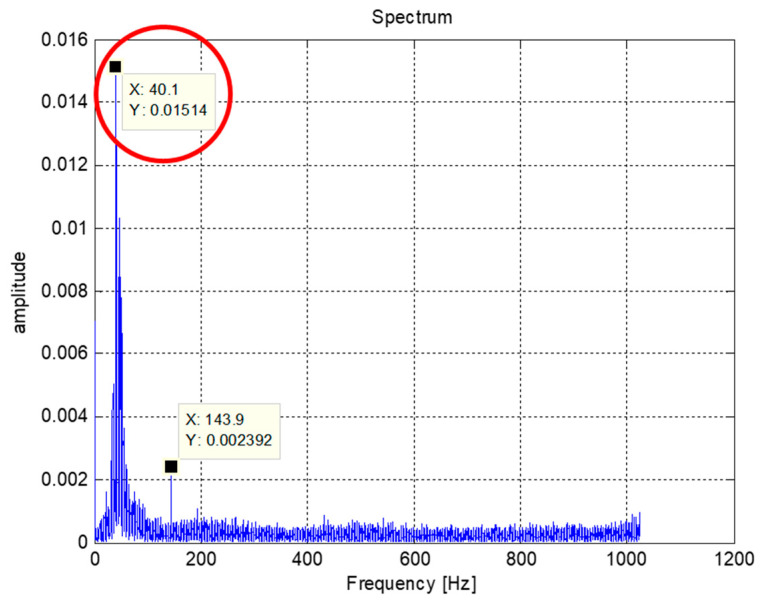
The frequency spectrum of the gear vibration signal; the meshing frequency at 40 Hz was observed.

Considering that the axis closest to the wafer affects the clamping action, this study focuses on the end mechanism to establishing an experimental platform for research and analysis. Figure 11 shows the wear plant form, which is manufactured and assembled with a motor, controller, and finished product. An AC servo motor and a computer numerical control (CNC) milling machine controller was used to simulate the actual operation of a robot arm, where the motor rotation was set at 60 rpm reciprocation 360° forward and reverse, and paused for 0.3 s at the end of both turns. The motor shaft rotated for 360°, and the output shaft rotated for 257° since the gear ratio was 40:56:56. The selected sensor, which was wireless data transmission, was provided by Microprogram Information Co. Ltd. As the sampling frequency was 2048 Hz, the max gear meshing frequency was 40 Hz simultaneously. Through the experimental measurement of the gear vibration signal, the frequency spectrum is shown in Figure 12, from which the meshing frequency was observed at about 40 Hz. It was confirmed that the frequency of the signal to be measured was within the measurement range.

### 4.2. Gear Wear Data Acquisition

**Figure 13:**
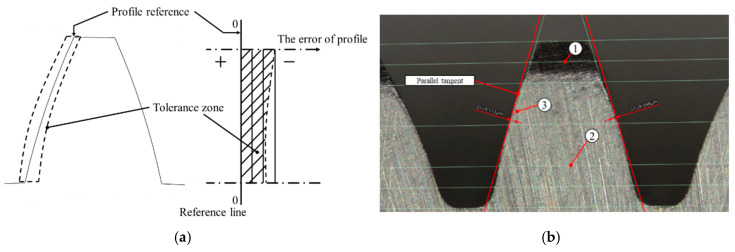
Measure of gear. (**a**) Industry gear profile tolerance [25]. (**b**) Measurement diagram; the distance of auxiliary line 3 to the parallel tangent is the wear amount.

In order to measure the physical quantity of gear wear, the newly unsealed gear was manually sanded with sandpaper for deburring, then the gear was photographed with an electron microscope Keyence VK-X1000 and the wear amount was measured by drawing auxiliary lines in the analysis software. The difference in the gear profile was defined as shown in Figure 13a. The auxiliary line 1 and 2 of the third midline for the key points of gear tip were drawn by following the definition of industrial gear profile tolerances [25]. The measured physical wear value was auxiliary line 3 to the parallel tangent to the profile, as shown in Figure 13b. In actual gear samples of the robot, the wear amount was 4.271 μm; and the average value of the gears on the experimental platform before and after wear were 27.463 μm and 23.217 μm, respectively, and the wear amount was about 3.464 μm.

To accelerate the degradation, the new gears that were deburred manually were to wear in about 30,000 rounds on the experimental platform, and the vibration signals of the entire wear process were collected. The radial vibration direction was reasonable and obvious on the physical characteristics, thus the *y*-axis signal was only selected. The numerical control (NC) code used for the experiment was as follows.

G90G54X0.F300.
#31 = 30,000
N1
IF[#31 <=0]GOTO2
G91 X +5. F300.
G04 X0.3
G91 X -5. F300.
G04 X0.3
#31 = #31-1
GOTO1
N2
M30

**Figure 14:**
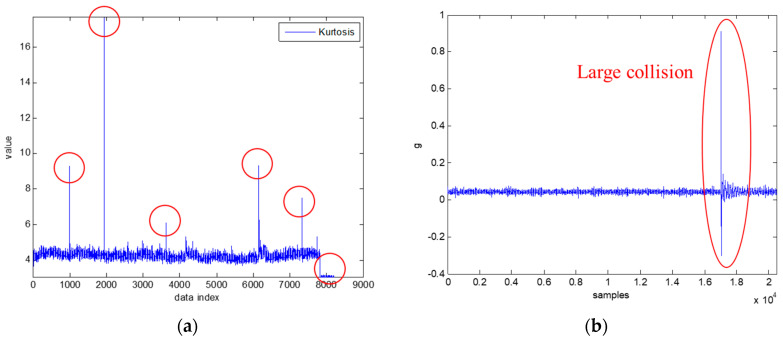
Data clearing illustration. (**a**) Kurtosis (abnormal data). (**b**) Raw signals.

There was rotation forward and reverse with a pause for 0.3 s in the middle as one loop. The sensor was set to store 10 s of data in length. As a result, about 15,600 files of vibration signals during a gear wear process were obtained. Before training, the data clearing was done to observe the statistical features in root mean square (RMS) and Kurtosis. Figure 14 shows the Kurtosis and raw data of each sample, respectively. It can be observed that the area selected by the red circle was abnormal data and standby time. The raw vibration signal is shown in Figure 14b, where there was an oscillation caused by a larger collision. The running-in problem of gears was also considered, furthermore, 1000 data were selected after running-in as OK, according to RMS and Kurtosis features.

### 4.3. Experimental Results

**Figure 15:**
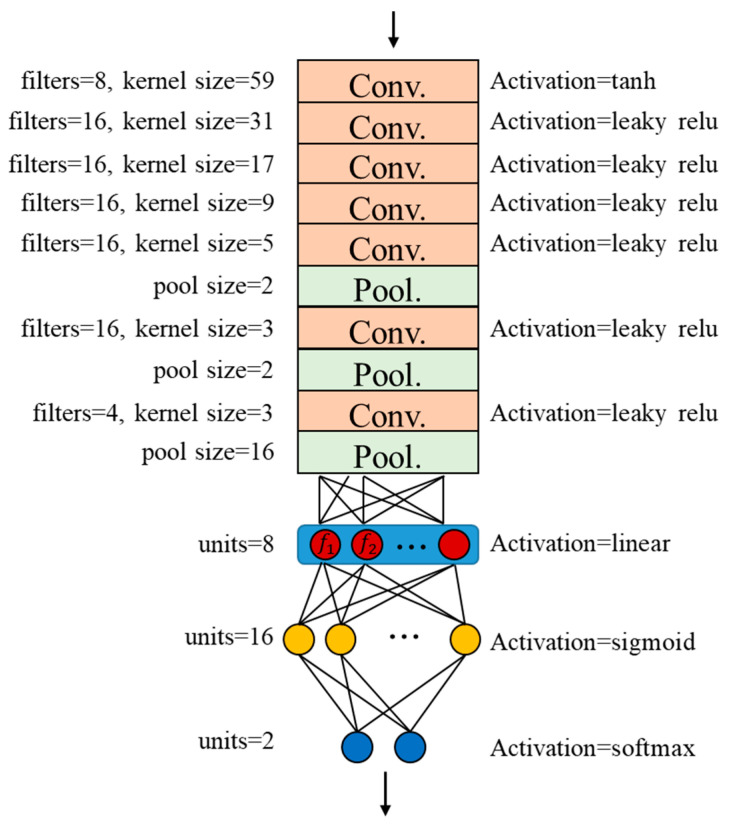
The detailed structure designed.

**Figure 16:**
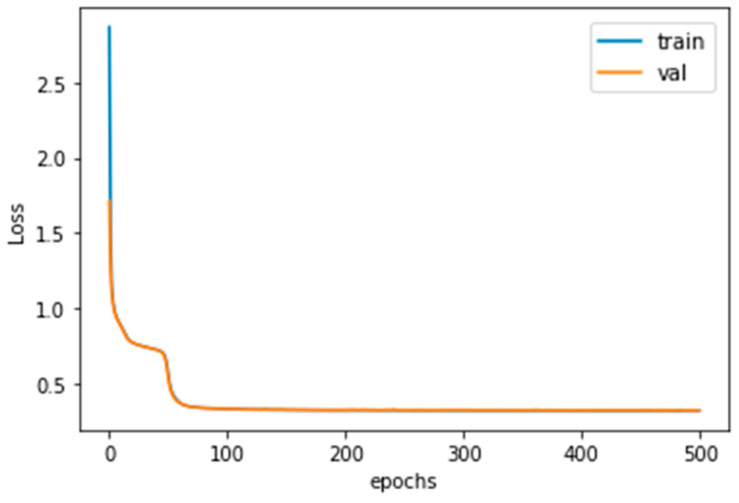
The training loss history.

**Figure 17:**
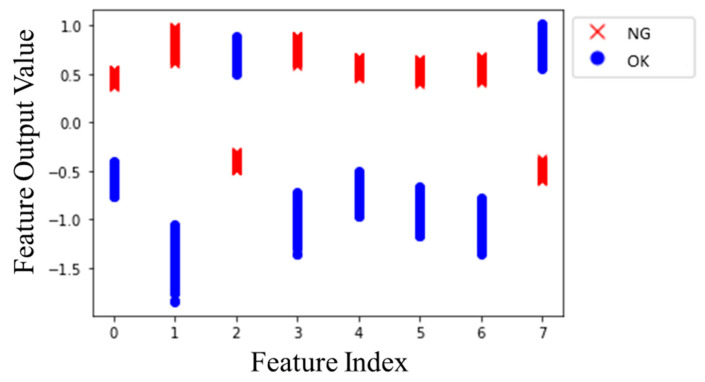
Feature clustering chart.

The model structure of the proposed method is shown in Figure 15. The length of the input vibration signal *L_s_* was 12,000, which was twice as long as the operating cycle; the time series input parameters were *N_s_* = 5 and *T_s_* = 6. The classifier part was designed as a fully connected simple neural network structure of [8,16,2] (one hidden layer). Finally, the estimation *P* was done by Equation (10), and *W* is the average wear amount of the experimental gears of 3.464. As above, the OK and NG training data were used to train the model. The corresponding learning parameters were introduced in Table 5, and the training results of the model is shown in Figure 16, it shows that the overfitting phenomenon is not serious. The final training loss and classification accuracy of each set are shown in Table 6. The training results of features are shown in Figure 17, and it could be observed that the feature outputs were divided into two clusters.

**Figure 18:**
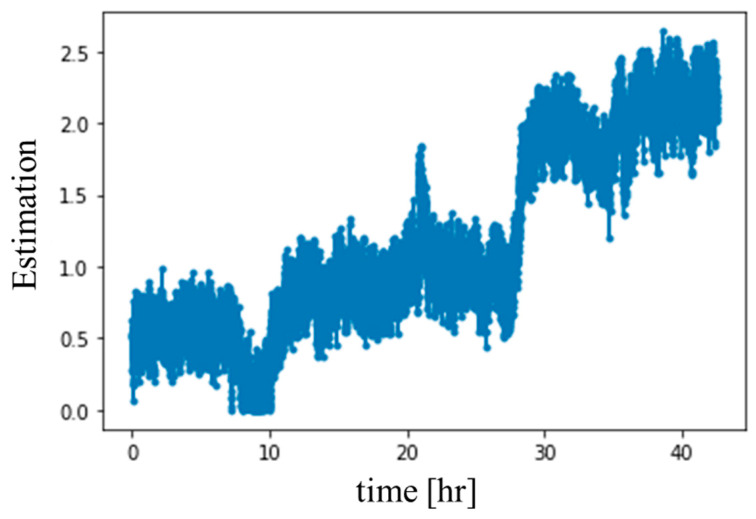
The continuous estimation chart.

In order to evaluate the estimation performance of the proposed method, the test gear entire wear process data were used for estimation. The estimated value was plotted with time as shown in Figure 18 to simulate obtaining pieces of data over time. As it can be seen that the variations in estimated values were positively correlated with wear time, and the final estimated and actual wear amounts were about 2.240 μm and 2.471 μm, respectively, which were the average values of 10 points. The estimation error was about 0.231 μm, which is about 10%, and shows the ability of the proposed method.

**Figure 19:**
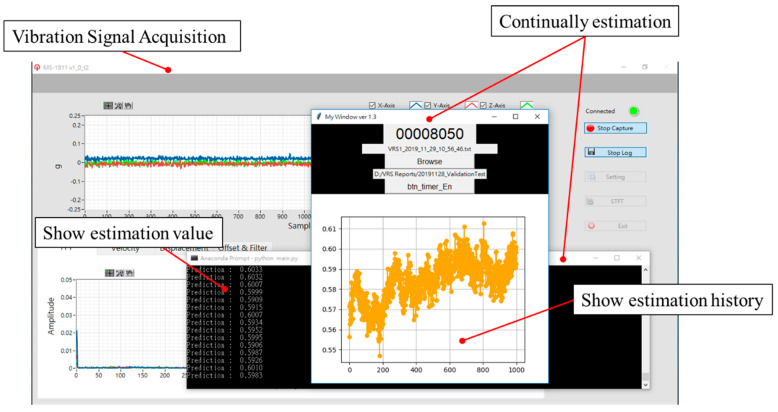
The continuous monitoring user interface program.

Figure 19 shows the user interface of a continuous monitoring program for prognosis gear wear by integrating the trained model and the proposed estimation method. It runs concurrently with the vibration sensor measurement program provided by the manufacturer of the sensors. When the program judges that the sensor has accessed a new vibration signal, it estimates the wear amount through the previous data, according to the parameters *N_s_*, *T_s_*, and *L_s_*.

## 5. Conclusions

This paper proposed a prognosis method by using 1-D CNN with hybrid loss functions in the absence of entire wear data. Referring to the other studies, the 1-D CNN model was used as a suitable characteristic and subjoined the time-series concept, combined with clustering loss. The features for prognosis were automatically extracted by a deep learning model instead of manual extraction and clustered into each category. Then, the open bearing dataset was used to validate the proposed method preliminarily. After a series of pre-processing data simulating the manufacturer’s lack of the entire wear process data, the proposed method was analyzed by designing different *N_s_* and *T_s_* and comparing the estimation error and functionality with other studies. Furthermore, a practical problem of gear wear was obtained for verification. An experimental platform was designed to simulate one of the axes on a semiconductor robot, and collected the vibration signals of the gear wear process, and measured the wear amount of the gears after the wear. As can be seen from the results, the variations in the estimated values were positively correlated with wear time, and the estimation error was about 0.231 μm (10%), which demonstrated the performance of the proposed method. In addition, a continuous monitoring program for prognosis gear wear was obtained by integrating the trained model and estimation method into a user interface program.

## Figures and Tables

**Table 1:** The training algorithm and parameters.

Algorithm and Parameters	Values
Learning algorithm	Adam
Initial learning rate	0.0001
Decay	0
Learning epochs	1000
Batch learning size	64
*α*	2
*β*	1

**Table 2:** The comparison results of popular algorithms.

	Adam	Rmsprop	Adagrad	Momentum	Gradient Descent
Initial learning rate	0.0001				
Epoch number	1000				
Clustering loss	**0.007**	0.012	0.099	0.061	0.130
Classify loss	**0.314**	0.314	0.694	0.693	0.694
Train data accuracy	**100.00%**	99.91%	10.13%	50.00%	50.00%
Val. data accuracy	**99.61%**	99.65%	12.11%	50.00%	50.00%
Test data accuracy	**91.29%**	50.00%	50.00%	50.00%	50.00%

**Table 3:** The classification accuracy comparison results for each trained model.

**Model**		**(2, 2)**	**(3, 2)**	**(4, 2)**	**(5, 2)**	**(6, 2)**	**(7, 2)**	**(8, 2)**
**Accuracy**	Training	99.96%	99.96%	99.92%	100.00%	99.92%	100.00%	99.92%
	Validation	99.48%	99.65%	99.83%	99.13%	99.65%	99.83%	99.83%
	Testing	92.00%	89.70%	91.23%	91.02%	91.39%	82.30%	86.43%
**Model**		**(2, 5)**	**(3, 5)**	**(4, 5)**	**(5, 5)**	**(6, 5)**	**(7, 5)**	**(2, 10)**
**Accuracy**	Training	100.00%	99.92%	99.96%	99.96%	100.00%	99.96%	100.00%
	Validation	99.83%	100.00%	100.00%	99.80%	99.80%	99.48%	99.48%
	Testing	91.93%	91.29%	91.29%	87.50%	91.36%	91.42%	88.78%
**Model**		**(3,10)**	**(4, 10)**	**(5, 10)**	**(6, 10)**	**(2, 20)**	**(3, 20)**	**(4, 20)**
**Accuracy**	Training	99.92%	99.96%	100.00%	99.91%	99.96%	99.96%	99.91%
	Validation	99.80%	99.61%	99.61%	100.00%	99.65%	100.00%	100.00%
	Testing	90.31%	91.10%	91.29%	87.40%	90.94%	92.09%	91.07%
**Model**		**(5, 20)**	**(2, 40)**	**(3, 40)**	**(4, 40)**	**(2, 60)**	**(3, 60)**	**(2, 80)**
**Accuracy**	Training	99.95%	99.96%	99.90%	99.89%	99.91%	99.89%	99.90%
	Validation	99.80%	99.80%	100.00%	100.00%	99.80%	100.00%	99.80%
	Testing	91.84%	94.36%	92.38%	87.28%	97.48%	90.91%	97.23%

**Table 4:** The clustering performance comparison.

	Without Clustering Loss	With Clustering Loss
*D*	174.0700	0.8884
*r*_0_	11.3940	0.0190
*r*_1_	122.7900	0.0326
*L_cluster_*(*D_norm_*)	0.1316	0.0071

**Table 5:** The training algorithm and parameters.

Algorithm and Parameters	Values
Learning algorithm	Adam
Initial learning rate	0.0001
Decay	0
Learning epochs	500
Batch learning size	64
*α*	2
*β*	1

**Table 6:** The training performance.

Performance	Values
Train data accuracy	100.00%
Val. data accuracy	100.00%
Test data accuracy	87.03%
Train data loss	0.318

## References

[1] Lee J., Wu F., Zhao W., Ghaffari M., Liao L., Siegel D. (2014). Prognostics and Health Management Design for Rotary Machinery Systems—Reviews, Methodology and Applications. Mech. Syst. Signal Process..

[2] Rangel-Magdaleno J., Peregrina-Barreto H., Ramirez-Cortes J., Cruz-Vega I. (2017). Hilbert Spectrum Analysis of Induction Motors for the Detection of Incipient Broken Rotor Bars. Measurement.

[3] Jin X., Sun Y., Que Z., Wang Y., Chow T.W. (2016). Anomaly Detection and Fault Prognosis for Bearings. IEEE Trans. Instrum. Meas..

[4] Zhang R., Gu F., Mansaf H., Wang T., Ball A.D. (2017). Gear Wear Monitoring by Modulation Signal Bispectrum Based on Motor Current Signal Analysis. Mech. Syst. Signal Process..

[5] Lei Y., Li N., Guo L., Li N., Yan T., Lin J. (2018). Machinery Health Prognostics: A Systematic Review from Data Acquisition to RUL Prediction. Mech. Syst. Signal Process..

[6] Guo L., Li N., Jia F., Lei Y., Lin J. (2017). A Recurrent Neural Network Based Health Indicator for Remaining Useful Life Prediction of Bearings. Neurocomputing.

[7] Zhao R., Yan R., Chen Z., Mao K., Wang P., Gao R.X. (2019). Deep Learning and Its Applications to Machine Health Monitoring. Mech. Syst. Signal Process..

[8] Yao Y., Wang H., Li S., Liu Z., Gui G., Dan Y., Hu J. (2018). End-to-end Convolutional Neural Network Model for Gear Fault Diagnosis Based on Sound Signals. Appl. Sci..

[9] Sohaib M., Kim C.-H., Kim J.-M. (2017). A Hybrid Feature Model and Deep-Learning-based Bearing Fault Diagnosis. Sensors.

[10] Hoang D.T., Kang H.J. (2019). Rolling Element Bearing Fault Diagnosis Using Convolutional Neural Network and Vibration Image. Cogn. Syst. Res..

[11] Min E., Guo X., Liu Q., Zhang G., Cui J., Long J. (2018). A Survey of Clustering with Deep Learning: From the Perspective of Network Architecture. IEEE Access.

[12] Aljalbout E., Golkov V., Siddiqui Y., Strobel M., Cremers D. (2018). Clustering with Deep Learning: Taxonomy and New Methods. arXiv.

[13] Hong S., Zhou Z., Zio E., Hong K. (2014). Condition Assessment for the Performance Degradation of Bearing Based on a Combinatorial Feature Extraction Method. Digit. Signal Process..

[14] Yang C., Wu T. (2015). Diagnostics of Gear Deterioration Using EEMD Approach and PCA Process. Measurement.

[15] Zhao H., Deng W., Yang X., Li X. (2016). Research on a Vibration Signal Analysis Method for Motor Bearing. Optik.

[16] Chen X., Feng F., Zhang B. (2016). Weak Fault Feature Extraction of Rolling Bearings Based on an Improved Kurtogram. Sensors.

[17] Sinha A. (2010). Vibration of Mechanical Systems.

[18] Ren S., He K., Girshick R., Sun J. (2015). Faster R-CNN: Towards Real-Time Object Detection with Region Proposal Networks. Advances in Neural Information Processing Systems.

[19] Krizhevsky A., Sutskever I., Hinton G.E. (2012). Imagenet classification with deep convolutional neural networks. Advances in Neural Information Processing Systems.

[20] Gu J., Wang Z., Kuen J., Ma L., Shahroudy A., Shuai B., Liu T., Wang X., Wang G., Cai J. (2018). Recent Advances in Convolutional Neural Networks. Pattern Recognit..

[21] Ince T., Kiranyaz S., Eren L., Askar M., Gabbouj M. (2016). Real-time Motor Fault Detection by 1-D Convolutional Neural Networks. IEEE Trans. Ind. Electron..

[22] Lo C.C., Chen B.S., Lee C.H. Fault Detection and Remaining Useful Life Estimation of Bearing Using Deep Learning Approach. Proceedings of the International Conference on Advanced Robotics and Intelligent Systems.

[23] Feng K., Borghesani P., Smith W.A., Randall R.B., Chin Z.Y., Ren J., Peng Z. (2019). Vibration-based updating of wear prediction for spur gears. Wear.

[24] Nectoux P., Gouriveau R., Medjaher K., Ramasso E., Chebel-Morello B., Zerhouni N., Varnier C. (2012). PRONOSTIA: An Experimental Platform for Nearings Accelerated Degradation Tests. Proceedings of the 2012 IEEE Conference on Prognostics and Health Management, PHM’12.

[25] Gimpert D. (2005). A New Standard in Gear Inspection. Gear Solutions.

